# *Vibrio cholerae* strains with inactivated *cqs*S gene overproduce autoinducer-2 which enhances resuscitation of dormant environmental *V*. *cholerae*

**DOI:** 10.1371/journal.pone.0223226

**Published:** 2019-10-01

**Authors:** Iftekhar Bin Naser, M. Mozammel Hoque, Shah Nayeem Faruque, M. Kamruzzaman, Shinji Yamasaki, Shah M. Faruque

**Affiliations:** 1 School of Life Sciences, Independent University Bangladesh, Dhaka, Bangladesh; 2 Department of Mathematics and Natural Sciences, BRAC University, Dhaka, Bangladesh; 3 Laboratory Sciences and Services Division, International Centre for Diarrhoeal Disease Research, Bangladesh, Dhaka, Bangladesh; 4 Department of Biochemistry and Microbiology, North South University, Dhaka, Bangladesh; 5 Department of Veterinary Science, Graduate School of Life and Environmental Sciences, Osaka Prefecture University, Osaka, Japan; Defense Threat Reduction Agency, UNITED STATES

## Abstract

**Background:**

Toxigenic *Vibrio cholerae* resides in aquatic reservoirs of cholera-endemic areas mostly in a dormant form known as conditionally viable environmental cells (CVEC) in which the bacteria remain embedded in an exopolysaccharide matrix, and fail to grow in routine bacteriological culture. The CVEC can be resuscitated by supplementing culture media with either of two autoinducers CAI-1 and AI-2, which are signal molecules controlling quorum sensing, a regulatory network of bacterial gene expression dependent on cell density. This study investigated possible existence of variant strains that overproduce AIs, sufficient to resuscitate CVEC in environmental waters.

**Methods:**

Environmental *V*. *cholerae* isolates and Tn insertion mutants of a *V*. *cholerae* strain C6706 were screened for production of AIs using bioluminescent reporter strains. Relevant mutations in environmental strains which overproduced AI-2 were characterized by nucleotide sequencing and genetic complementation studies. Effect of AIs produced in culture supernatants of relevant strains on reactivation of CVEC in water was determined by resuscitation assays.

**Results:**

Two of 54 environmental *V*. *cholerae* isolates were found to overproduce AI-2. Screening of a Tn-insertion library of *V*. *cholerae* strain C6706, identified a mutant which overproduced AI-2, and carried Tn insertion in the *cqsS* gene. Nucleotide sequencing also revealed mutations inactivating the *cqsS* gene in environmental isolates which overproduced AI-2, and this property was reversed when complemented with a wild type *cqsS* gene. Culture of river water samples supplemented with spent medium of these mutants resuscitated dormant *V*. *cholerae* cells in water.

**Significance:**

*V*. *cholerae* strains with inactivated *cqsS* gene may offer a convenient source of AI-2 in enhanced assays for monitoring bacteriological quality of water. The results also suggest a potential role of naturally occurring *cqsS* mutants in the environmental biology of *V*. *cholerae*. Furthermore, similar phenomenon may have relevance in the ecology of other waterborne bacterial pathogens beyond *V*. *cholerae*.

## Introduction

*Vibrio cholerae* as a species naturally inhabits estuarine or fresh water environments, although toxigenic strains of the species infect humans and may cause epidemics of cholera, which is a devastating diarrheal disease [1, 2]. In the aquatic environment, *V*. *cholerae* exists mostly in a dormant form referred to as viable but non-culturable (VBNC), alternatively known as conditionally viable environmental cells (CVEC) [3], a metabolic state that is believed to enhance the survival of nonsporulating bacteria in an adverse environment [4].

In Bangladesh, where cholera is endemic, *V*. *cholerae* can be readily detected in water in a culturable form, particularly during seasonal epidemics of cholera [5]. However, during the inter-epidemic period, the organisms are mostly found in the CVEC state [3], and special culture techniques or fluorescent antibody based microscopy are required to detect such dormant cells [3, 6, 7]. Previously, we demonstrated that CVEC are organized as dense aggregates of cells embedded in an extracellular polysaccharide matrix [8]. The genes responsible for this *Vibrio* extracellular polysaccharide production are controlled by two autoinducers (CAI-1 and AI-2) and their cognate receptors CqsS and LuxP respectively which are components of an intricate regulatory network of bacterial gene expression dependent on cell density, referred to as quorum sensing [9–12].

We recently showed that autoinducers CAI-1 and AI-2 enhance CVEC resuscitation, a process that can be controlled and the precise effect of added autoinducers on resuscitation of CVEC in water can be studied [6]. However, we found that the activity of autoinducers required to effectively resuscitate CVEC in the laboratory was notably higher than that found in culture supernatants of typical *V*. *cholerae* strains [6], and hence, the source of possible exogenous autoinducers involved in natural resuscitation of dormant *V*. *cholerae* in environmental waters, remains unclear. In the present study we explored the possibility that certain genetic variants of *V*. *cholerae* might overproduce one or more autoinducers, which might be sufficient to cause resuscitation of CVEC in water. In addition to providing further knowledge of the environmental biology and epidemiology of *V*. *cholerae*, such variant strains could be a useful source of exogenous autoinducers for potential application in improving laboratory diagnostics, including detection of dormant bacteria in water samples.

## Materials and methods

### Bacterial strains and culture condition

Relevant characteristics of various strains, and genetic constructs used in this study are listed in Table 1. The transposon insertion library of *Vibrio cholerae* strain C6706 used in this study has been described previously [13]. Other genetically marked strains or mutants used were either obtained from our collection or were constructed during the present study as described later.

**Table 1:** Description of bacterial strains and plasmids included in this study.

Strain/plasmid	Relevant Genotype	Description
pJZ176	*cqsA* gene cloned in pTAC under an IPTG inducible *lac* promoter	Recombinant plasmid carrying CAI-1 synthase gene
pJZ365	*luxS* gene cloned in pTAC under an IPTG inducible *lac* promoter	Recombinant plasmid carrying AI-2 synthase gene
p*cqsS*	*cqsS* gene cloned in pUC18	Recombinant plasmid carrying *cqsS* gene
DH5α (pJZ176)	*E*. *coli* DH5α carrying pJZ176	Produces CAI -1 on IPTG induction
DH5α (pJZ365)	*E*. *coli* DH5α carrying pJZ365	Produces AI-2 on IPTG induction
C6706	A *lacZ* negative *V*. *cholerae* O1 El Tor strain used to construct the Tn insertion library	*V*. *cholerae* O1 El Tor biotype strain
IMGL-11	C6706, Δ*cqsA*	Deficient in CAI-1
EC20699	C6706,*luxS*::TnFGL3	Deficient in AI-2
IMGL-12	C6706,*luxS*::TnFGL3 Δ*cqsA*	Deficient in both CAI-1 and AI-2
EC2635	C6706,cqsS::TnFGL3	Deficient in sensor for CAI-1
EC2635-1	EC2635 (pCqsS)	cqsS mutant carrying a cloned cqsS gene
EC1547	C6706,luxP::TnFGL3	Deficient in sensor for AI-2
MGL-13	C6706,luxP::TnFGL3 cqsS::cat	Deficient in both AI-1 and AI-2 sensors
VC3625; VC987	Environmental *V*. *cholerae* O1 variants carrying mutations inactivating *CqsS* gene	Hyperproducers of AI-2
MM920	*V*. *cholerae* Δ*cqsA*Δ*luxQ* (pBB1). Plasmid pBB1 carries the *luxCDABE* operon of *V*. *harveyi*	Produces bioluminescence specifically in response to CAI-1
BB170	*V*. *harveyi* luxN::Tn5	Produces bioluminescence specifically in response to AI-2

Environmental *V*. *cholerae* strains were isolated from surface water in Dhaka, Bangladesh using an enrichment culture technique referred to as AST described previously [7]. Briefly, an aliquot (5.0 ml) of each water sample was added to 2.5 ml of 3X concentrated bile peptone medium (BP, 1% peptone, 0.5% taurocholic acid, 1% NaCl, pH 9.0), and incubated for 5h for enrichment of *V*. *cholerae*. Dilutions of the enrichment cultures were spread on taurocholate tellurite gelatine agar (TTGA) [14] plates containing streptomycin (70 μg/ml). Suspected *Vibrio* colonies were picked and subjected to standard biochemical and serological tests as described previously [7]. Strains used in this study were routinely cultured in Luria-Bertini broth (LB) or LB-agar containing appropriate antibiotics at 37° C, unless otherwise stated.

### Construction of deletion mutants

Deletion mutants were constructed using a method described by us previously [6]. Briefly, two short DNA segments flanking the two ends of the target gene were amplified by PCR. A fusion product was prepared by ligating the amplified fragments, and the fused product was further amplified using forward primer of the left flanking fragment and the reverse primer of the right flanking fragment. The suicide vector pRE112 was used to clone the PCR amplified fragment and *E*. *coli* SM10λpir was transformed with the recombinant plasmid, and further grown. The recombinant plasmid isolated from *E*. *coli* was then electroporated into appropriate recipient *V*. *cholerae* strains and plated on LB agar plates containing chloramphenicol (20 μg/ml). Colonies were counter selected by growing in LB with 5% sucrose as described previously [6], to identify the ones which lost the resistance marker and therefore the suicide vector. Suspected mutants were further subjected to PCR analysis to confirm the expected genotype.

### Chitin induced transformation

*V*. *cholerae* cells were transformed in the presence of chitin substrate as described previously [15, 16] to create defined insertion mutants by marker exchange. In this process, DNA containing the mutated gene was obtained from an appropriate Tn insertion mutant in the TnFGL3 insertion library [13]. Briefly, overnight cultures of the recipient *V*. *cholerae* strains were diluted in LB medium and grown to an OD_600_ of ≈0.3. The bacterial pellet collected by centrifugation was washed, and resuspended in a 0.10 volume of 0.5% sterilized environmental water. Aliquots of bacterial suspension (2 ml) were dispensed into a 12-well tissue culture plate containing pieces of sterile shrimp shell, and incubated at 30°C, without shaking for 24 h. The aqueous phase was then removed, and fresh water containing 1–2 μg of the appropriate DNA was added to the wells. After another 24 h of incubation, the pieces of shrimp shells were removed, washed, and vortexed to release attached bacteria which were then plated on LB-agar containing an appropriate antibiotic to select transformants. Appropriate PCR based analysis was done to further analyze the suspected transformants, and confirm mutations by marker exchange.

### Preparation of cloned AIs

Autoinducers were prepared from cultures of *E*. *coli* DH5α carrying cloned genes for CAI-1 and AI-2 synthase. The recombinant constructs pJZ176 and pJZ365 kindly provided as a gift by Jun Zhu (Department of Microbiology, University of Pennsylvania School of Medicine, Philadelphia) respectively carried the *cqsA* and *luxS* genes cloned in pTAC under an IPTG inducible *lac* promoter as described previously [17]. *E*. *coli* DH5α carrying the recombinant plasmids were grown overnight in LB medium containing ampicillin (50μg/ml). The culture was diluted in fresh LB medium containing the antibiotic and grown for a further 3–4 h (until OD_600_ = 0.5–1.0). The culture was centrifuged at 4500 x g for 15 min to precipitate cells. The supernatant which contained antibiotic was discarded, the cell pellet was resuspended in original volume of fresh LB medium without antibiotic, and IPTG solution was added to a final concentration of 0.5mM. The culture was incubated at 37°C with shaking for another 6–8 h, and the culture supernatant was collected after precipitating cells by centrifugation. The supernatant which contained autoinducer was further sterilized by filtration through 0.22μm pore-sized filters as described previously [17].

### Preparation of autoinducer using mutant *V*. *cholerae* strains

*V*. *cholerae*, strain *C6706ΔcqsAΔluxS (pcqsA)* or *C6706ΔcqsAΔluxS (pluxS)* which carried recombinant plasmids with cloned genes for synthesis of CAI-1 and AI-2 respectively instead of their respective indigenous autoinducer synthase gene was grown in LB medium at 37°C with shaking for 16-18h. The culture was centrifuged to precipitate cells, and the supernatant which contained AI expressed from the recombinant plasmids was collected and filter-sterilized using 0.22μm pore-sized filters.

### Bioluminescence assay for CAI-1 and AI-2 activity

Cell-free supernatants of *V*. *cholerae* or *E*. *coli* cultures were the source of autoinducers, and the activity of CAI-1 and AI-2 were assayed by inducing light production in two bioluminescence reporter strains MM920 and BB170 respectively as described previously [18–20]. Briefly, single colonies of the respective strains were inoculated into 5 ml LB medium and incubated overnight at 37°C with shaking. The cultures were then diluted 1:100 in fresh LB and grown to the same optical density (OD_600_ = 1.5). Cultures were centrifuged to precipitate cells, and the supernatants were filtered through a 0.22μm pore sized filter. This cell-free culture supernatants were tested for the presence of CAI-1 or AI-2 activity as follows. The reporter strain was grown overnight with shaking at 30°C, diluted 1:10 in fresh LB medium, and 70 ml aliquots were transferred to an opaque-wall 96-well microtitre plate. The cell-free culture fluids of the source strains were added to a final concentration of 30% (v/v). The plates were incubated at 30°C with agitation, and light production was measured at 30 min intervals in a GeniosPlus plate reader. Bioluminescence was expressed in Relative light units (RLU) defined as counts min^-1^ ml^-1^ x 10^3^/CFU ml^-1^.

### DNA sequencing

Nucleotide sequencing was performed using an automated DNA sequencing system (ABI Prism 310; PE Applied Biosystems, Foster City, CA) with BigDye terminator cycle sequencing ready reaction kit (PE Applied Biosystems). Initially, subclones of the *cqsS* gene from relevant strains were constructed in pUC19, and were sequenced using universal sequencing primers (Invitrogen). The nucleotide sequence of both strands of *cqsS* were further determined by primer walking with primers derived from the preliminary sequencing of *cqsS* subclones in pUC19. Sequences were processed using the Sequencher alignment program, Version 4.0 (Gene Codes Corporation, Inc., MI). The nucleotide sequence of *cqsS* derived from different strains were compared to sequences in the GenBank databases using the National Centre for Biotechnology Information BLAST server program.

### Resuscitation of *V*. *cholerae* O1 CVEC in water

Environmental water samples which were selected for this assay were initially found to be negative for *V*. *cholerae* O1 in conventional enrichment cultures. Three ml of water was added to 3 ml LB medium and 3 ml of the AI preparation, and incubated at 37°C with shaking. At regular intervals, aliquots of the culture were withdrawn and plated on TTGA [14] containing an appropriate antibiotic [7]. As control assays, samples without adding the AI preparation were also incubated and analyzed in parallel. At the same time, aliquots of the same water samples were subjected to fluorescent-labeled antibody based detection of *V*. *cholerae* O1 as described previously [21]. Briefly, bacterial cells concentrated from aliquots of water samples by centrifugation were stained using fluorescein isothiocyanate-conjugated *V*. *cholerae* O1-specific antiserum, and examined under UV light using an epifluorescence microscope equipped with a digital camera for recording images.

### Statistical analysis

The build-in data analysis program in Microsoft Excel (MS office version 2007) was used to conduct general statistical analysis of data, and were expressed as mean ± standard deviation. The differences were tested by two-tailed t-test, and values of P < 0.05 were considered statistically significant.

### Institutional approvals

All experimental protocols were approved by the Research Review Committee (RRC) and the Ethics Review Committee (ERC) of the International Centre for Diarrhoeal Disease Research, Bangladesh (Protocol numbers PR-15029 and PR-07018). All methods were conducted in accordance with the guidelines of the RRC and ERC.

## Results

### Identification of environmental *Vibrio cholerae* strains overproducing AI-2

**Fig 1:**
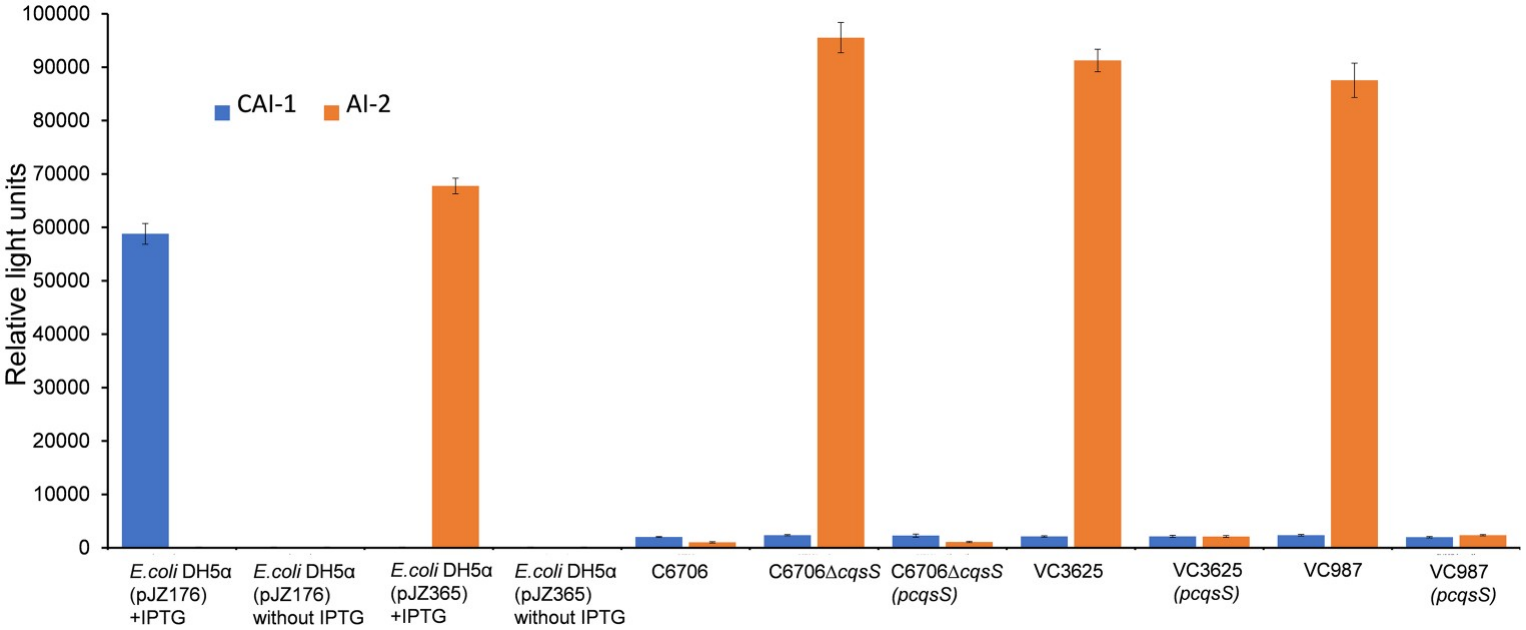
Activity of autoinducers produced by different bacterial strains and their derivatives as assayed using bioluminescence produced by reporter strains for CAI-1 and AI-2 (see text for details). VC3625 and VC987 are environmental *V*. *cholerae* O1 strains whereas C6706 is a laboratory strain of *V*. *cholerae* O1. Recombinant plasmids pJZ176 and pJZ365 carry CAI-1 and AI-2 synthase genes respectively, whereas pcqsS carries a cloned *cqsS* gene which encodes the sensor for AI-2.

Autoinducers such as CAI-1 and AI-2 have been shown to act as resuscitation factors for dormant forms of *V*. *cholerae* present in water samples, when assayed in the laboratory [6]. In order to look for possible environmental *V*. *cholerae* strains which might overproduce CAI-1 or AI-2, we analyzed 54 isolates of *V*. *cholerae* O1 or non-O1 using two reporter strains MM920 and BB170 respectively [18–20]. These reporter strains (Table 1) responded specifically to the respective autoinducers present in the spent culture supernatant of different strains by expressing bioluminescence [19, 20]. In these assays we also included as controls, culture supernatants of recombinant *E*. *coli* strains carrying cloned genes for CAI-1 and AI-2 synthesis under an IPTG inducible promoter, as well as a *V*. *cholerae* O1 laboratory strain C6706 (Fig 1). We identified 2 *V*. *cholerae* O1 strains designated as VC3625 and VC987 both of which vastly overproduced AI-2 in their spent culture supernatants (Fig 1). The level of AI-2 produced by these strains was ~100 fold more than that produced by strain C6706 whereas that of CAI-1 production was similar to that of the control strain C6706. We further investigated possible genetic changes that might have led to the observed hyperproduction of AI-2 by these two environmental strains, as described later.

### Genetic lesions associated with overproduction of AI-2

To characterize the nature of likely genetic alterations that would result in increased production of one or more autoinducers we screened a transposon insertion library [13] of the El Tor biotype *V*. *cholerae* O1 strain C6706, to identify possible mutants whose spent culture supernatant would show high levels of CAI-1 or AI-2 when tested using the reporter strains MM920 and BB170 respectively [19, 20].

In this screen we identified a mutant showing the desired property, and remarkably like the two environmental strains which overproduced AI-2, this mutant also overproduced AI-2 as evident from increased bioluminescence produced by the AI-2 reporter strain (Fig 1). The activity of AI-2 in the culture supernatant of this mutant was also about 100 fold higher than that produced by the parent strain C6706 (p = 0.0002). The mutant strain designated EC2635 (16) carried TnFGL3 insertion inactivating the *cqsS* gene which encodes the receptor for the autoinducer CAI-1. None of the other mutants in the Tn insertion library showed this property. Notably, complementation with a wild type *cqsS* gene in a recombinant plasmid p*cqsS* abolished the ability of the strain to overproduce AI-2 (Fig 1). Taken together these observations suggested that strains sustaining mutational inactivation of the *cqsS* gene encoding the CAI-1 sensor overproduce AI-2.

### Environmental strains overproducing AI-2 carry naturally inactivated *cqsS* gene

**Fig 2:**
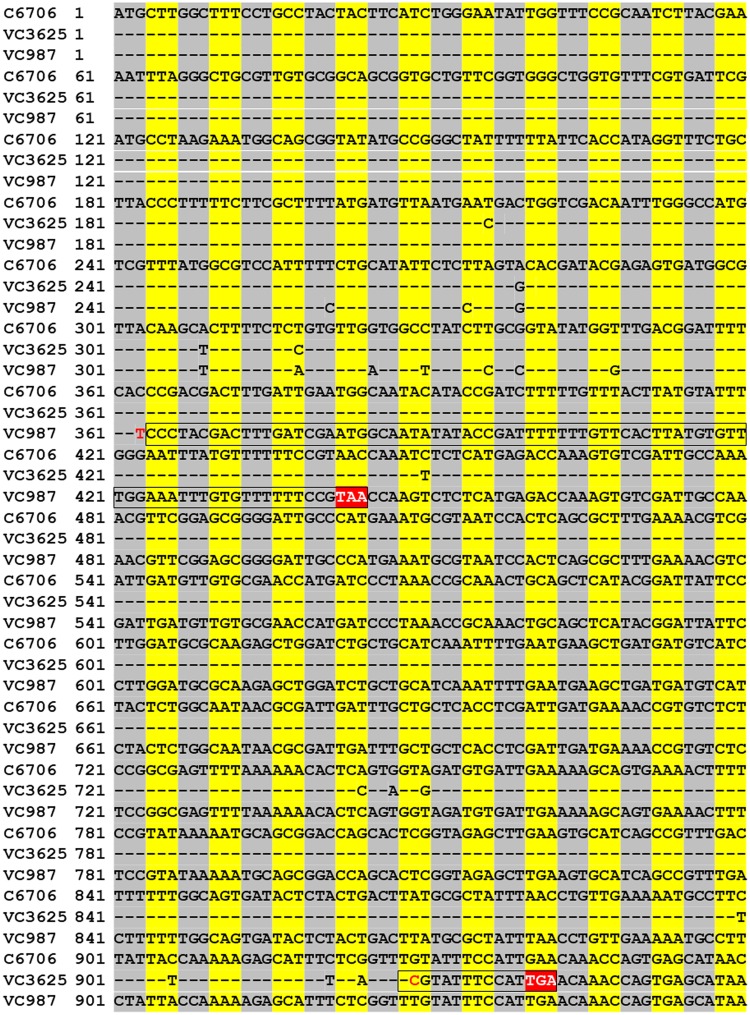
Alignment of the nucleotide sequences of the *cqsS* gene of strain C6706 with that of two environmental *V*. *cholerae* strains VC3625 and VC987 carrying spontaneous mutations in the *cqsS* gene and overproducing AI-2. The sequence of the coding region from 1 to 960 bases are shown. In the two environmental strains VC3625 and VC987, the *cqsS* genes are shown to have sustained spontaneous nucleotide insertions at position 353 and 929 respectively (highlighted in red font) resulting in frame shift (black box), and introduction of a stop codon (highlighted in red).

To investigate whether like the Tn insertion mutant of C6706 the two environmental strains which overproduce AI-2 sustained mutational inactivation of the cqsS gene, we sequenced PCR amplicons of the *cqsS* gene in these strains and compared with that of strain C6706 [22]. This analysis confirmed a number of substitutions and insertions in the *cqsS* gene in these two environmental strains. The nucleotide insertions in both these strains, albeit at different positions caused frame shifts that had effectively inactivated the gene (Fig 2). Moreover, like the laboratory generated mutants of strain C6706, complementation with a plasmid clone of the wild type *cqsS* gene abolished the ability of these environmental strains to overproduce AI-2 (Fig 1). These results supported our assumption that the observed hyper production of AI-2 arose through spontaneous mutations leading to inactivation of the *cqsS* gene in these strains.

### Culture supernatants of *cqsS* mutants resuscitate CVEC in water samples

Previously, we showed that autoinducers CAI-1 and AI-2 resuscitated dormant *V*. *cholerae* known as CVEC, which exist in environmental waters [6]. Since culture supernatants of *V*. *cholerae* strains carrying mutations in *cqsS* gene were found to contain high levels of AI-2 using the reporter strain, we examined whether these culture supernatants also enhanced resuscitation of CVEC in water samples. The samples were collected in aquatic sites in Dhaka, Bangladesh, since similar water samples had yielded evidence of CVEC in a previous study [6].

Aliquots of water were initially tested by enrichment and culture for the presence of *V*. *cholerae* as described previously [7] to exclude samples which might contain a small proportion of culturable *V*. *cholerae* cells. For resuscitation assays with various spent medium (Table 2), only water samples that were negative for *V*. *cholerae* in the initial enrichment culture were included. Addition of 50% vol:vol spent medium from the strains carrying inactivated *cqsS* gene was found to cause resuscitation of dormant *V*. *cholerae* O1 cells in many water samples within a few hours of enrichment. Resuscitation of *V*. *cholerae* O1 was also noticed in the presence of spent culture supernatants of various *E*. *coli* or *V*. *cholerae* strains expressing either CAI-1 or AI-2 from recombinant plasmids carrying the respective genes. When enriched in an identical manner but exposed to 50% vol:vol spent media of control cultures, aliquots of these same water samples were mostly found negative for culturable *V*. *cholerae*. The spent medium used in the control assays, included those of C6706,*luxS*::TnFGL3Δ*cqsA* which was a derivative of *V*. *cholerae* strain C6706 with deletion of its indigenous AI synthase genes, and *E*. *coli* DH5α carrying the empty cloning vector pTAC. Similarly, spent culture supernatants of *cqsS* deleted strains harboring a recombinant plasmid carrying the wild type *cqsS* gene failed to show resuscitation of dormant *V*. *cholerae* in the water samples (Table 2).

**Table 2:** Recovery of dormant *V*. *cholerae* O1 CVEC in environmental water by culturing in medium supplemented with spent culture supernatant of various bacterial strains and genetic constructs.

Date of sampling	No. of samples tested^b^	Number of samples found positive for *V*. *cholerae* O1 after enrichment in LB containing filter- sterilized spent medium^a^ of different strains										
		(A) C6706	(B) DH5α (pTAC)^c^ +IPTG	(C) DH5α (pJZ176)^c^ +IPTG	(D) DH5α (pJZ364)^c^ +IPTG	(E) *C6706ΔcqsAΔluxS*	(F) *C6706ΔcqsS*	(G) *C6706ΔcqsS* (pcqsS)	(H) VC3625	(I) VC3625 (pcqsS)	(J) VC987	(K) VC987 (pcqsS)
Aug-14	11	1	0	8	5	1	8	2	9	0	6	1
Sep-14	10	2	0	7	5	0	6	0	5	1	7	3
Oct-14	12	0	0	7	4	0	6	0	8	0	5	0
Nov-14	15	0	1	7	5	0	7	0	8	1	7	0
Dec-14	12	2	0	6	4	1	9	1	5	1	7	0
Jan-15	11	1	0	6	5	0	8	1	6	2	4	1
Feb-15	15	0	1	6	4	0	9	0	7	0	6	0
Mar-15	12	2	0	5	4	0	6	3	5	0	5	2
Apr-15	10	1	1	7	6	0	5	1	7	1	4	2
Total samples	108	9	3	59	42	2	64	8	60	6	51	9
% of total	100	8.3	2.7	54.6	38.8	1.8	59.2	7.4	55.5	5.5	47.2	8.3

^a^The composition of enrichment media are shown. Samples were inoculated into LB media supplemented with 50% (v/v) spent medium of the indicated strain and then incubated for 6 hours at 37°C, before plating aliquots of the enrichment culture on TTGA plates containing streptomycin and SXT.

^*b*^Only CVEC positive surface water samples are shown

^*c*^*pJZ176* and *pJZ364* are plasmids carrying cloned *cqsA* and *luxS* genes respectively cloned in *pTAC*.

The observed differences in recovery of culturable cells when enriched under different conditions were statistically significant (p <0.001) when compared between columns: B vs C, B vs D, A vs C, A vs D, F vs G, H vs I, and J vs K.

As we reported previously, in the resuscitation assays there was no recovery of cells in the first 3 to 4 hours of incubation of various samples, whereas viable cells appeared suddenly within 4–5 hours of exposure to the spent medium which contained an autoinducer. This dynamics of observed cell count was not consistent with possible multiplication of a low number of culturable cells by binary fission, but instead suggested resuscitation of a large number of dormant cells.

### Morphological changes associated with CVEC resuscitation

**Fig 3:**
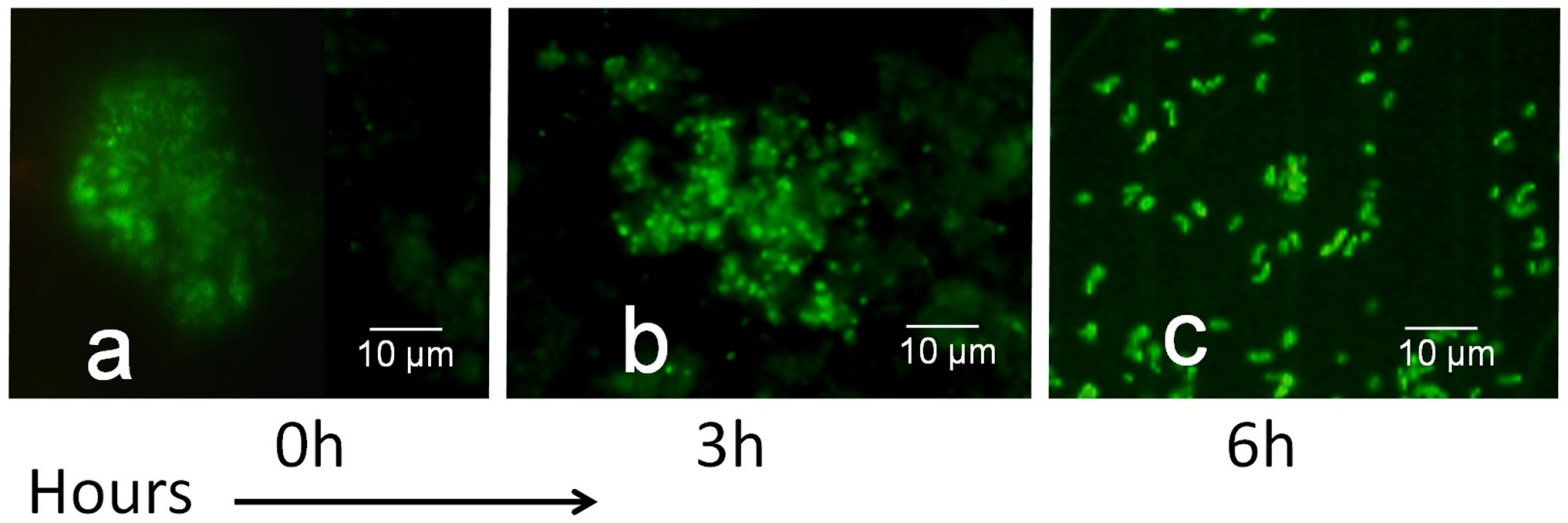
Fluorescent antibody based microscopic visualization of the CVEC resuscitation process. Multiple aliquots of water samples were incubated in LB medium containing equal volumes of cell-free spent culture supernatant of an AI-2 overproducing strain VC3625. Aliquots of samples were removed at regular time intervals, concentrated by centrifugation and monitored using fluorescent antibody based microscopy. Morphology of *V*. *cholerae* O1 CVEC at different time intervals of the resuscitation process are shown (panel a through c). In these samples, the morphology of cells changed gradually from a coccoid shape to more rod-like shape. These cellular changes corresponded to the recovery of viable cells at 6 hours of incubation.

While culturable *V*. *cholerae* cells are mostly rod-shaped the dormant form of *V*. *cholerae* known as CVEC have been previously reported to be typically clumps of cells with a somewhat coccoid morphology and plenty of slimy exopolysaccharides that is distinctly different from cells present in exponentially growing cultures [6]. We observed morphological changes in possible CVEC present in water samples during the resuscitation process by fluorescent anti-O1 antibody-based examination (Fig 3). In cultures exposed to AI preparations, within 3h of incubation, cells began to disperse although still remained somewhat coccoid in shape (Fig 3, panel b). However, the cells became culturable by 6h of incubation, and these aggregates transformed from a coccoid to more rod-like shapes. We also attempted to grow cells from the water samples after mechanical disruption to disperse possible CVEC by vortexing vigorously with silica beads at different time points without adding autoinducer preparations. However, mechanical disruption did not produce viable counts at T = 0 or 3h and 6h without autoinducer treatment. Thus, resuscitation is not just related to dispersion of clumped cells but involves additional metabolic activities. As shown previously [6] these changes in cell morphology and the gradual disappearance of the extracellular polysaccharide matrix correlated with the detection of culturable cells after exposure to media containing autoinducers.

## Discussion

We showed previously that dormant *V*. *cholerae* referred to as CVEC present in water samples could be resuscitated in the laboratory by supplementing the enrichment culture media with either of two autoinducers CAI-1 or AI-2 [6]. In this previous study we found that the level of autoinducer normally produced by a typical *V*. *cholerae* O1 strain such as C6706 in the culture supernatant was not enough to induce the resuscitation, but required considerably more autoinducer as produced by cloning autoinducer synthase genes. The results of the present study represent significant advancement on this topic in that we have identified and characterized spontaneous mutants that can naturally produce significantly high level of an autoinducer. We identified naturally occurring strains which produce about 100 fold more AI-2 than typical strain C6706, and their culture supernatants could be directly used in resuscitation assays for CVEC in water samples. In our previous study (6), we showed that recombinant *E*. *coli* DH5α strains carrying the cloned AI-2 synthase gene produced sufficient level of AI-2 to cause resuscitation of CVEC in environmental water samples. In the present study we found that the naturally occurring *V*. *cholerae* variant strains produced even higher levels of AI-2 than the recombinant *E*. *coli* strains (Fig 1). We conducted a number of mutational analysis of the *V*. *cholerae* strain C6706 to demonstrate that genetic variants that overproduce AI-2 carry inactivated *cqsS* gene which encodes a receptor for another autoinducer CAI-1. Remarkably, the naturally occurring AI-2 hyper-producing variants were also found to carry a spontaneously inactivated *cqsS* gene.

The precise genetic pathway that lead to overproduction of AI-2 in these variants is not clear at this time, and was not within the scope of the present study. Nevertheless, these results clearly suggest the existence of a regulatory mechanism in *V*. *cholerae* to boost AI-2 production, when the major autoinducer CAI-1 is not sensed by the cells presumably due to a lack of its receptor.

It is well known that some bacterial populations seem to “disappear” from natural water bodies during certain times of the year, only to reappear at other times, and that such “disappearance” occurs due to their entry into a dormant physiological state [2–4]. We previously showed that the dormant state is linked to biofilm formation by bacteria [8] and indeed such cells respond to autoinducers which are signal molecules that lead to dispersion of biofilm associated cells [3, 6]. Notably, the hyperproduction of AI-2 by *cqsS* mutants is presumably driven by a need for high levels of AI-2 when the other autoinducer CAI-1 is not sensed by the cells due to mutational inactivation of its sensor. The precise regulatory mechanisms which lead to this phenotype would be an area for further investigations in quorum sensing pathways.

The results of this study also provide several other new insights into the ecological and epidemiological features of *V*. *cholerae*. Because AI-2 molecules can be produced and sensed by multiple bacterial species, it follows that such AI-2 over-producing strains might lead to sudden emergence of viable cells of *V*. *cholerae* or other bacterial pathogens from any environment that is replete in AI-2 molecules. Our study further suggest that the CVEC could resuscitate, due to their exposure to AI-2 molecules produced by various environmental bacterial species or by the intestinal microbiota present in humans or animals. However, while AI molecules produced by normal human gastrointestinal microbiota might also cause resuscitation of CVEC inside the human host ingesting such dormant cells, the seasonality of cholera in geographical areas like the Ganges Delta might involve multiple other factors. For example, if seasonal environmental parameters such as temperature, salinity, carbon content etc., give rise to blooms of heterologous bacterial organisms in the environment and if a portion of these are spontaneous hyper-producers of AI-2 signals, then dormant pathogenic bacteria in the vicinity e.g., *V*. *cholerae* might be resuscitated and more readily cause human disease during this period.

It may also be important to understand the parameters that allow *V*. *cholerae* to produce its own resuscitation factors, since wild type *V*. *cholerae* C6706 did not produce spent medium that was significantly active in resuscitation assays on CVEC (Table 1). Since the bacterial population in the environment could represent a complex habitat where differentiation occurs, we presume that a proportion of environmental cells might undergo mutations to become hyper-producers of one or more autoinducers and thus trigger the resuscitation of other dormant cells. Our results support this assumption since we found that indeed several environmental strains of *V*. *cholerae* possess naturally occurring frame shift mutations in the *cqsS* gene, and these strains are hyperproducers of AI-2. Moreover, complementation with the wild type cqsS gene abolished this property (Fig 1). Notably, these variant strains did retain the functional gene encoding the receptor for AI-2.

It seems possible that seasonality of waterborne bacterial diseases such as cholera may be influenced by bacterial interspecies communication. Further ecological studies might, therefore, provide novel means for surveillance, prevention and control of cholera. Numerous other pathogenic bacteria have been shown to enter into the dormant state after exposure to adverse environmental stresses [4, 23, 24]. These include *E*. *coli*, *V*. *vulnificus*, *Shigella sonnei*, *S*. *flexneri*, *Salmonella enteritidis*, *Campylobacter jejuni*, and *Legionella pneumophila*. Therefore, understanding natural mechanisms for the emergence of variant strains overproducing resuscitation factors, such as AI-2 may have implication in understanding the epidemiology of diseases caused by various other bacterial species besides *V*. *cholerae*.

From an applied standpoint, effective monitoring of bacteriological quality of water constitutes an important step in understanding transmission of waterborne diseases, and the risk of outbreaks. Currently available culture techniques are inadequate to detect bacterial pathogens which fail to grow in routine media. Therefore, use of autoinducers to enhance bacterial resuscitation may open up possibilities to develop modified techniques for routine use in testing bacteriological quality of water samples. In particular, use of AI-2 to supplement culture media would potentially resuscitate multiple species of bacteria which might exist in water in a dormant form. We predict that our results would form a strong basis for continuing studies in multiple areas of environmental biology of waterborne bacterial pathogens, and improvement in testing bacteriological quality of water.
